# Localized Surface Phonon Polaritons and Infrared Optical Absorption of ScAlN Nanoresonators

**DOI:** 10.3390/ma18163906

**Published:** 2025-08-21

**Authors:** Huanhuan Zhao, Tao Cheng, Xinlei Duan, Mingxin Lv, Jia-Yue Yang, Linhua Liu

**Affiliations:** 1 School of Nuclear Science, Energy and Power Engineering, Shandong University, Jinan 250061, China; zhaohh@mail.sdu.edu.cn; 2 Optics & Thermal Radiation Research Center, Institute of Frontier and Interdisciplinary, Shandong University, Qingdao 266237, China; tao_c@mail.sdu.edu.cn (T.C.); 202321157@mail.sdu.edu.cn (X.D.)

**Keywords:** surface phonon polaritons, infrared optical absorption, dielectric function, polar crystal

## Abstract

Alloying AlN with ScN provides a robust strategy for engineering its intrinsic bandgap, phonons and dielectric functions, and ScAlN alloys have demonstrated great promise in applications including the 5G mobile network, surface acoustic wave devices and nanophotonics. Sc doping has been shown to greatly influence the phonons and infrared dielectric functions of AlN, yet few studies have focused on its influence on surface phonon polaritons, which are crucial to modulating the radiative properties of ScAlN metasurfaces. Herein, we combined first-principles and finite element method (FEM) simulations to fully investigate the effects of Sc incorporation on the phonon dispersion relation, propagation and localization of SPhPs and the modulated radiative properties of ScAlN nanoresonators. As the Sc doping concentration increases, the highest optical phonon frequencies are reduced and are largely directly related to enlarged lattice parameters. Consequently, the coupling strength among incident photons and phonons decreases, which leads to a reduced absorption peak in the infrared dielectric functions. Moreover, the propagation length of the SPhPs in ScAlN is largely reduced, and localized resonance modes gradually disappear at a higher Sc doping concentration. This work provides physical insights into the spectra tuning mechanisms of ScAlN nanoresonators via Sc doping and facilitates their applications in nanophotonic devices.

## 1. Introduction

Scandium aluminum nitride (ScAlN) exhibits an extraordinary piezoelectric response, spontaneous polarizability and ferroelectricity, which advocate for it as a promising candidate in fields such as high-electron-mobility transistors and ferroelectric and surface acoustic wave devices [1,2,3,4,5]. Owing to its large bandgap, robust bandgap tunability via alloying and good compatibility with complementary metal oxide semiconductor (CMOS) fabrication technology, ScAlN also emerges as an active material for integrated photonics operating from to the UV to the mid-IR spectral range [6,7,8,9]. An in-depth exploitation of the light–matter interactions in ScAlN is relevant to accelerating photonic device design.

Dielectric function, as one of the fundamental thermophysical properties of matter, reflects the strength of light–matter interactions. Exploring the infrared dielectric function of ScAlN can help us understand the infrared phonon modes and their interactions with incident photons, which determine the optical absorption. Deng et al. [10] prepared a ScAlN thin film deposited on sapphire (0001) and measured the Raman and infrared reflectance spectra to extract the infrared phonon modes and investigate the effects of various Sc doping concentrations further. Mock and co-workers [11] grew wurtzite Sc_x_Al_1-x_N (0 ≤ x ≤ 0.2) thin films using the molecular beam epitaxy method and employed spectroscopic ellipsometry to measure their infrared dielectric functions. The measurements showed that the infrared dielectric function is largely modified and the phonons shift to lower frequencies with increasing incorporation of scandium. With substantial modulation of the infrared dielectric functions through Sc doping, the light–matter interactions, especially in ScAlN nanophotonics, would also differ largely. For pure AlN, the surface phonon polaritons (SPhPs) lying between the Reststrahlen band contribute significantly to engineering the absorption spectra by introducing nanoresonator structures [12]. In GaN/AlN heterostructures, the optical phonon modes are greatly modulated and a new Reststrahlen band emerges, which provides the opportunity to tune the infrared properties [13,14]. Also, tunable spectral behavior can be achieved through hybridization of the doping-dependent surface phonon–plasmon resonances in AlN/SiC heterostructures integrated with graphene [15]. Recently, SPhPs and infrared spectral modulation in polar crystals, including GaN [16], SiC [17] and SrTiO_3_ [18], have been fully explored. Though alloying AlN with Sc at a low concentration retains its polar nature, the behaviors of SPhPs in ScAlN are subtly different from those in AlN, mainly due to modification of the infrared dielectric function and phonon modes. Thus, it is of great necessity to explore the effects of Sc doping on the light–matter interactions in ScAlN-based nanophotonics.

In this work, we systematically investigate the influence of Sc doping on the infrared phonon modes, dielectric function and SPhPs of ScAlN and the localized resonances and absorption spectra of corresponding nanoresonators using first-principles and finite element method (FEM) simulations. The first-principles calculations indicate that the highest optical phonon frequencies are reduced by increasing Sc doping. As a consequence, the interactions among the incident photons and phonons become weakened, and the maximum value of the infrared dielectric function decreases, which reduces the propagation length of the SPhPs further. FEM simulations show that the resonance modes gradually disappear at a high Sc doping concentration, and the radiative properties of ScAlN nanoresonators can be robustly modulated by the structural geometry.

## 2. The Computational Methodology

### 2.1. The Lattice Structure and Phonons

**Figure 1:**
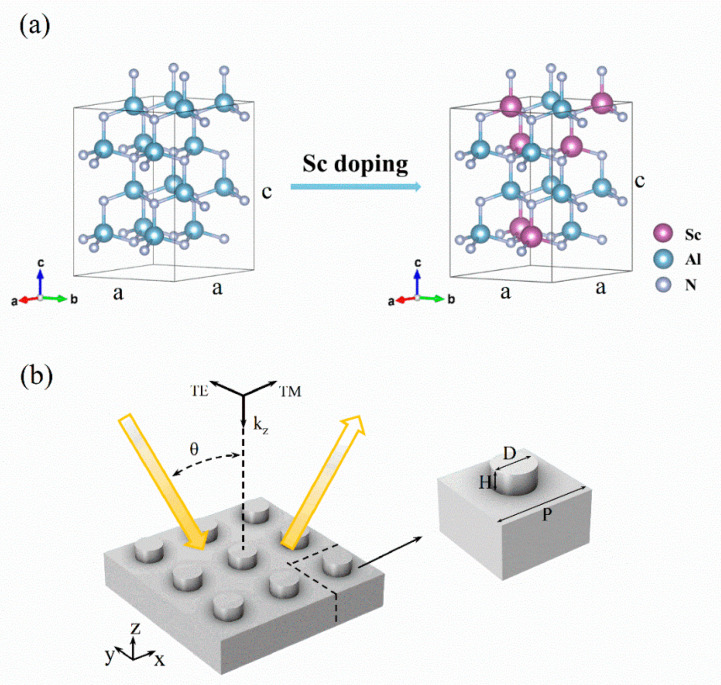
(**a**) Crystal structure transition of AlN and ScAlN with Sc doping and (**b**) schematic and parametric diagram of ScAlN nanoresonator structure defined by period (P), diameter (D) and height (H).

Under ambient conditions, AlN possesses a non-centrosymmetric wurtzite (space group, P6_3_mc) structure, while ScN exhibits a meta-stable layered hexagonal structure [19]. When doping AlN with a low Sc concentration (typically up to 35%), ScAlN retains the wurtzite lattice structure but is a disordered solid solution, as shown in Figure 1a. The special quasi-random structure (SQS) method [20] was adopted to simulate a disordered ScAlN supercell containing 16 atoms, and density-functional-theory-based first-principles simulations were performed using the Vienna ab initio simulation package (VASP) [21,22]. The Perdew–Burke–Ernzerhof (PBE) functional [23,24] was used to describe the exchange-correlation potential for Al, Sc and N atoms, and the plane-wave cutoff energy was set as 800 eV [23,24]. First-principles calculations demonstrate that the lattice constant *a* of ScAlN gradually increases with an increasing Sc concentration, while *c* varies slightly for a Sc doping concentration up to 40% [25]. For instance, the optimized lattice constants *a* and c of Sc_0.05_Al_0.95_N are 3.152 Å and 5.024 Å, while they are 3.221 Å and 5.047 Å for Sc_0.2_Al_0.8_N, which are in good agreement with previous experimental data [26,27]. With the relaxed lattice structure, the infrared phonon modes of ScAlN were calculated using the Phonopy package [28]. The finite displacement method was used to compute the interatomic force constants, and a large supercell containing 128 atoms was chosen.

### 2.2. Dielectric Function and Finite Element Simulations

With varying Sc doping concentrations, the intrinsic infrared photon–phonon interactions in ScAlN change significantly and thus influence the electromagnetic interactions of light with ScAlN-based nanoresonators. The infrared dielectric functions of ScAlN thin films grown through molecular beam epitaxy were measured using spectroscopic ellipsometry [11] and chosen as the thermophysical input for finite element method (FEM) simulations of ScAlN-based nanoresonators. In Figure 1b, a schematic diagram of a representative ScAlN nanoresonator is presented, and its geometry is defined by the period (P), diameter (D) and height (H). A transverse magnetic (TM) wave was launched within the xz plane at an incident angle of 0° (normal incidence *k_z_*) in the simulation. Floquet periodic boundary conditions were imposed in the X and Y directions for the FEM simulation to simulate the nanoresonator array structure, while perfectly matched layers with a thickness of a 1/3 of a wavelength were applied in the Z direction. The global convergence grid was set to 1/10 of a wavelength, and the grid of the nanoresonator was refined to 0.1 μm.

## 3. Results and Discussion

### 3.1. The Phonon Dispersion Relation of ScAlN

**Figure 2:**
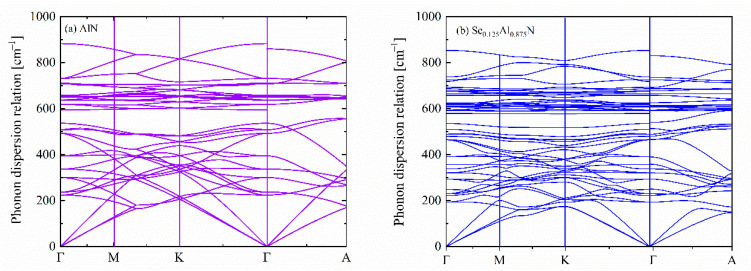
A comparison of the theoretically calculated phonon dispersion relation for (**a**) AlN and (**b**) Sc_0.125_Al_0.875_N from first-principles calculations. The high-symmetry-point coordinates are Γ (0,0,0), M (0.5,0,0), K (0.3333,0.3333,0) and A (0,0,0.5).

Sc incorporation alters the lattice structure of AlN and thus affect its lattice dynamic properties. Figure 2 compares the calculated phonon dispersion relations of AlN and Sc_0.125_Al_0.875_N. The non-existence of negative phonon modes in Sc_0.125_Al_0.875_N indicates its dynamical stability. With doped Sc, it is found that the highest frequency for the optical phonon decreases accordingly. For instance, the highest phonon frequency for AlN is 882.65 cm^−1^ but is reduced to 853.14 cm^−1^ for Sc_0.125_Al_0.875_N. Such a decrease in the phonon frequency is directly related to an enlarged lattice parameter. Equally, along the high-symmetric Γ-A path in the first Brillouin zone, the low-lying longitudinal acoustic phonons are softened by the doped Sc atoms.

### 3.2. The Dielectric Function and Surface Phonon Polaritons

**Figure 3:**
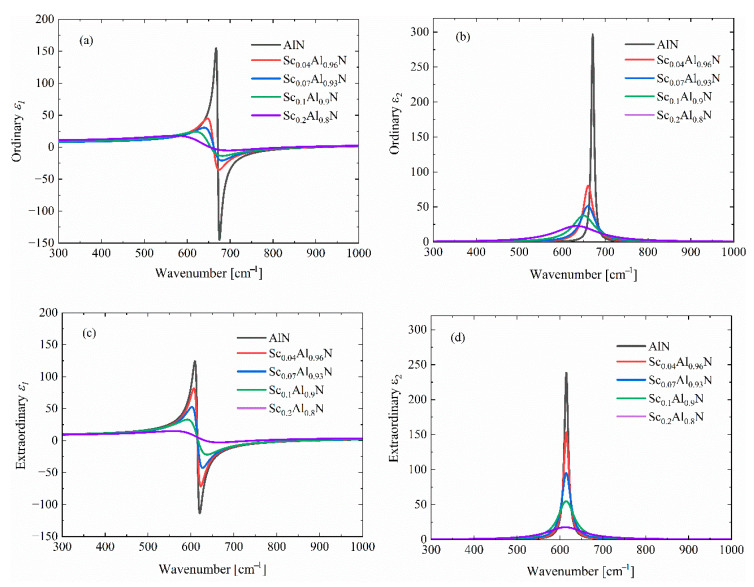
The (**a**,**b**) ordinary (⊥c) and (**c**,**d**) extraordinary (∥c) complex dielectric functions of ScAlN thin films under ambient conditions measured by Ref. [11].

The infrared dielectric function arises from intrinsic photon–phonon interactions, and phonon modes altered by Sc doping influence the dielectric function further. In Figure 3, the anisotropic complex dielectric functions of ScAlN with varying Sc concentrations under ambient conditions are presented and extracted from Ref. [11]. Owing to its wurtzite lattice structure, the ordinary (⊥c) dielectric function of ScAlN is largely different from that in the extraordinary (∥c) direction. As the doped Sc concentration increases, it is observed that the complex dielectric function is significantly altered. For instance, the maximum of the ordinary ε_2_ (see Figure 3a) is greatly reduced at a Sc doping concentration up to 20%; the width is largely broadened; and the location of the absorption peak shifts to a lower photon energy.

To quantify the effect of doping on the anisotropic dielectric function of ScAlN, the classical LO-TO oscillator model is used to fit the data from [29,30]:

(1) $$\varepsilon (\omega )=\varepsilon_{\infty }\frac{\omega^{2}-i\Gamma_{LO}\omega -\omega_{LO}^{2}}{\omega^{2}-i\Gamma_{TO}\omega -\omega_{TO}^{2}},$$

where *ω_TO_* and *ω_LO_* are the frequencies of the longitudinal and transverse optical phonons, respectively; *ε_∞_* is the high-frequency dielectric constant; and *Γ* is the broadened width. The fitted *ω_TO_* and *ω_LO_* values for the ordinary and extraordinary dielectric functions of ScAlN are presented in Table 1. For the ordinary dielectric function, both *ω_TO_* and *ω_LO_* decrease as the Sc doping concentration increases, while *ω_LO_* is largely reduced, but *ω_TO_* remains constant for the extraordinary direction.

**Figure 4:**
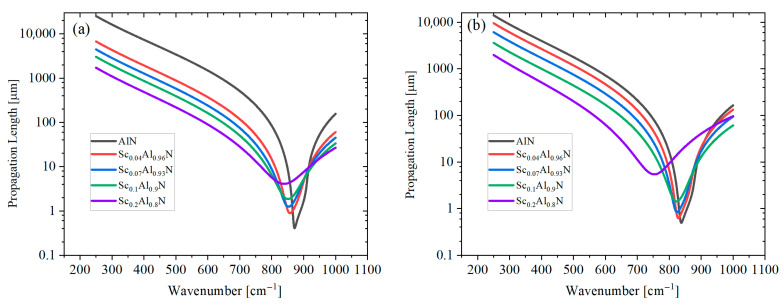
The SPhP propagation length of ScAlN in the (**a**) ordinary and (**b**) extraordinary directions with varying Sc doping concentrations.

Moreover, for incident photons with frequencies lying between *ω_TO_* and *ω_LO_*, it is found that the real part *ε*_1_ of the dielectric function is negative. These incident photons will propagate along the surface of ScAlN instead of being transmitted into it and couple with the lattice vibrations, thus forming surface phonon polaritons (SPhPs) [31,32,33,34]. The spectral range lying between *ω_TO_* and *ω_LO_* is defined as the Reststrahlen band (RB), where the incident photons are highly reflected by the ScAlN’s surface. With an increasing Sc doping concentration, the RB’s width is reduced, indicating that fewer incident photons can excite SPhPs. The intrinsic propagation length of the SPhPs propagating along the ScAlN’s surface is defined as Λ = (2Im *k*_sp_)^−1^ [35,36], where *k*_sp_ is a complex wave vector defined by *k*_sp_ = *ω*/*c*(*ε*/(1 + *ε*))^1/2^. *ε* is the complex dielectric function, and this relation applies to the single air–ScAlN interface. Figure 4a shows the SPhP propagation length in the ordinary direction at varying Sc doping concentrations. The propagation length initially decreases with an increasing wavenumber, exhibits a dip near 850 cm^−1^ and then increases at higher wavenumbers. Below 800 cm^−1^, increasing the Sc doping concentration results in a reduced propagation length. A similar trend is observed for the extraordinary direction, as shown in Figure 4b, although the overall propagation lengths are shorter compared to those in the conventional case. Equally, for Sc_0.1_Al_0.9_N, the highest propagation length can reach over 1000 μm, closely related to the long lifetime of optical phonons.

### 3.3. Localized SPhPs in ScAlN Nanoresonators

**Figure 5:**
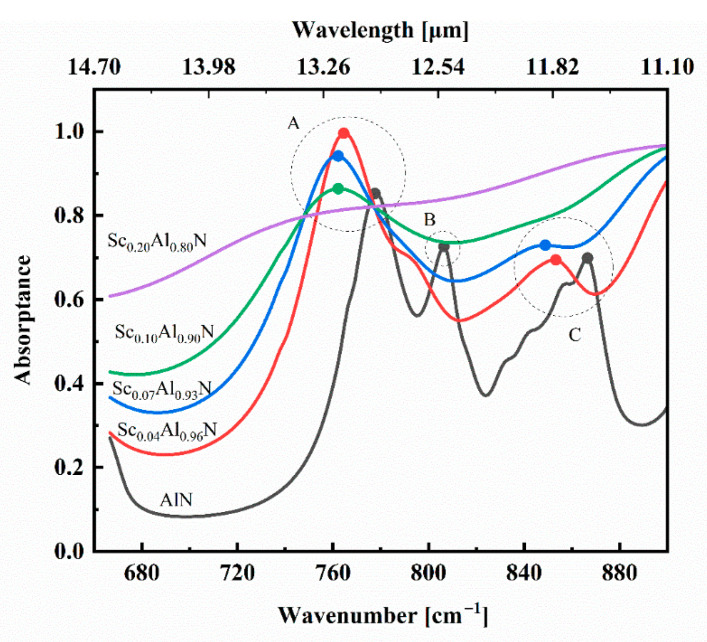
The calculated absorption spectra of the Sc_x_Al_1-x_N (0 ≤ x ≤ 0.2) nanoresonator at a normal incident angle for the TM wave.

**Figure 6:**
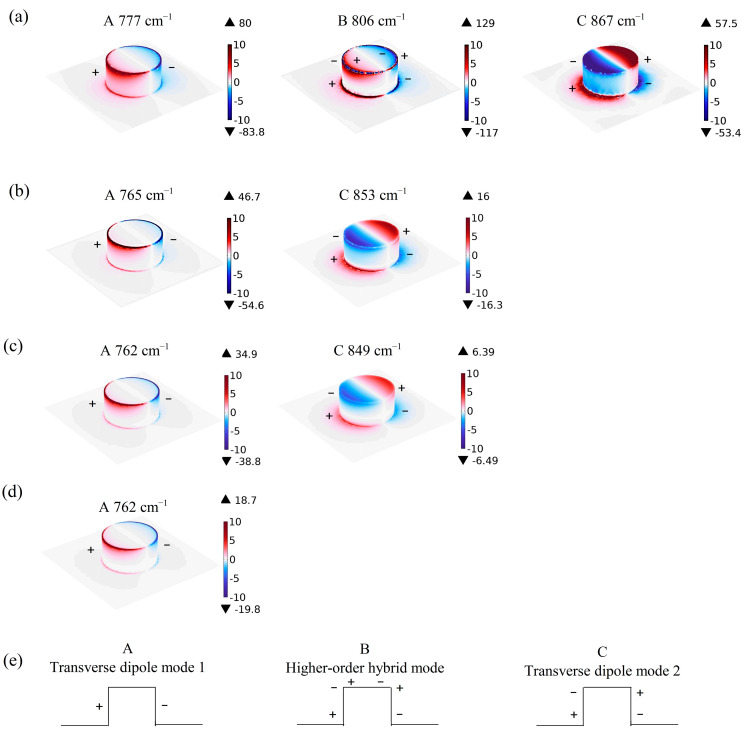
The charge density of (**a**) AlN at mode A (777 cm^−1^), mode B (806 cm^−1^) and mode C (867 cm^−1^); (**b**) Sc_0.04_Al_0.96_N at mode A (765 cm^−1^) and mode C (853 cm^−1^); (**c**) Sc_0.07_Al_0.93_N at mode A (762 cm^−1^) and mode C (849 cm^−1^); and (**d**) Sc_0.10_Al_0.90_N at mode A (762 cm^−1^) and (**e**) the schematic charge configurations of the three modes.

With such a large propagation length of the SPhPs in ScAlN, the introduction of nanostructuring onto the surface will induce localized surface phonon resonances and thus greatly modulate the optical properties. We calculated the absorption spectra of a Sc_x_Al_1-x_N (x = 0, 0.04, 0.07, 0.10 and 0.20) nanoresonator with P = 7 μm, D = 3 μm and H = 1.5 μm. In Figure 5, it is obvious that pure AlN exhibits three absorption modes: mode A (777 cm^−1^), mode B (806 cm^−1^) and mode C (867 cm^−1^). By analyzing the electric field distribution further, we find that modes A and C are excited by the mirror-symmetric in-plane electric field, and mode B is the higher-order hybrid mode, as shown in Figure 6. However, resonance modes B and C gradually disappear with an increasing Sc doping concentration. For Sc_0.04_Al_0.96_N, only modes A and C are observed, while only one mode, mode A, remains when the Sc incorporation increases to 10%, and no mode is observed at 20% doping. Moreover, from Figure 5, it is obvious that upon Sc doping, the phonons in the AlN films are shifted to lower frequencies, and simultaneously, the peak widths increase drastically.

**Figure 7:**
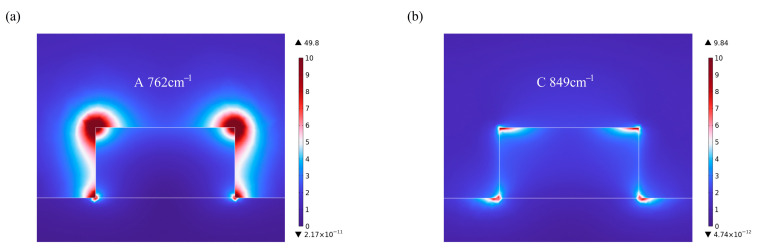
The normalized |E|-field distributions for the two absorption peaks at (**a**) A 762 cm^−1^ and (**b**) C 849 cm^−1^.

To display the relationship between the two absorption peaks (A and C in Figure 5) and the structural parameters intuitively, taking the Sc_0.07_Al_0.93_N nanoresonator as an example, the normalized |E|-field distributions of the two resonant wavenumbers (762 cm^−1^ and 849 cm^−1^) are calculated. From Figure 7a, it is observed that the |E|-field of absorption peak A (762 cm^−1^) is mainly concentrated around the nanoresonator, especially on the topside surface, while the second absorption peak C (849 cm^−1^) is mainly concentrated at the four corners of the nanoresonator. Additionally, peak C shows a much lower field intensity, indicating that the localization of this mode is relatively weak. And changes in the diameter and height of the ScAlN nanoresonator will affect the absorption spectra.

**Figure 8:**
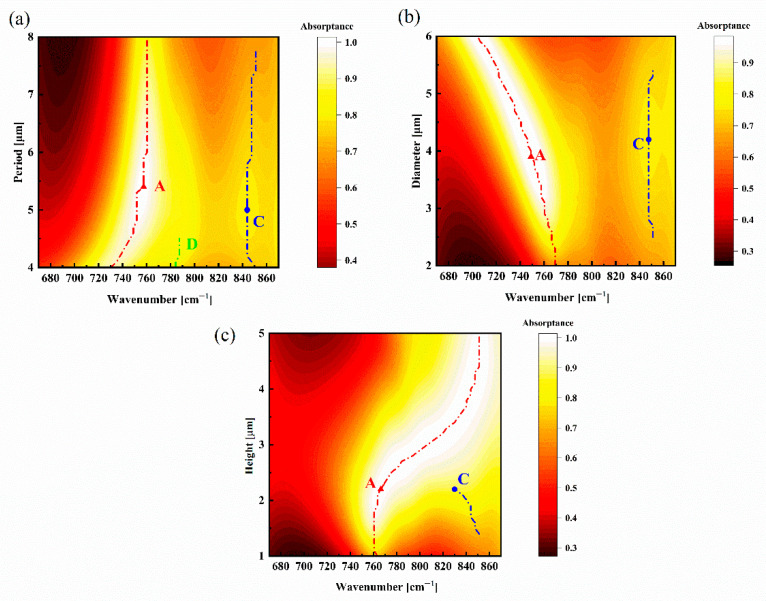
Tailoring the mode A (TD1) and mode C (TD2) resonance modes of a Sc_0.07_Al_0.93_N nanoresonator with varying structural parameters of (**a**) P, (**b**) D and (**c**) H at a normal incident angle for a TM electromagnetic wave.

Furthermore, we studied the absorption characteristics of a Sc_0.07_Al_0.93_N nanoresonator with varying geometrical parameters (4 μm ≤ P ≤ 8 μm, 2 μm ≤ D ≤ 6 μm and 1 μm ≤ H ≤ 5 μm). Figure 8a shows the absorption spectra pertinent to different periods P with D = 3 μm and h = 1.5 μm. The position of mode A (TD1)’s absorption peak blueshifts almost linearly (Δk ≈ 20 cm^−1^/ΔP = 0.6 μm) as P increases. But almost no change is observed for the two modes when P ≥ 6.0 μm. It is obvious that a new mode D (TD1) appears at k ≈ 785 cm^−1^ and is coupled with mode A (TD1) when P = 4.6 μm, which leads to greater spreading of mode A. However, after this point, the peak widths decrease drastically with increasing period. Figure 8b,c display the absorption spectra as the diameter varies with P = 7 μm and H = 1.5 μm and the height with P = 7 μm and D = 3 μm varies. Mode A exhibits an obvious redshift and blueshift with an increasing height and diameter, especially for height; when H = 1.4 μm, mode C appears and is coupled with mode A at H = 2.2 μm, while the position of mode C’s absorption peak shifts slightly with an increasing diameter. As the diameter increases, the peak widths of the two modes decrease gradually but increase drastically with increasing height. These correspond to the field distribution analysis in Figure 7.

By gradually changing the period, diameter and height, we find that the resonant amplitude is gradually enhanced with increasing structural parameters until it reaches the maximum value. As the height increases, the two modes progressively couple into broadband absorption modes, with this effect becoming particularly pronounced when H ≥ 2.2 μm.

## 4. Conclusions

To summarize, we have investigated the effects of the Sc doping concentration and geometric morphology on the infrared phonons, SPhPs and absorption spectra of ScAlN using first-principles calculations and FEM simulations. The incorporation of Sc into AlN will enlarge the lattice parameters and decrease the highest optical phonon frequencies. The absorption peak of the infrared dielectric function shifts to a lower wavenumber, and its amplitude decreases at a high Sc doping concentration, mainly due to weakened photon–phonon interactions. ScAlN can host SPhPs at low Sc doping concentrations, and their propagation length is over 1000 μm, providing great avenues for modulating the localized resonance modes and absorption spectra of nanoresonators. The geometric morphology also determines the localized SPhPs and absorption spectra. These findings could help exploit potential application scenarios for ScAlN metasurfaces as mid-IR photodetectors and thermal emitters.

## Figures and Tables

**Table 1:** The fitted frequency of LO-TO phonons and the corresponding Reststrahlen band (RB) width.

Sc_x_Al_1-x_N	Ordinary Dielectric Function			Extraordinary Dielectric Function		
	*ω_TO_* [cm^−1^]	*ω_LO_* [cm^−1^]	RB Width [cm^−1^]	*ω_TO_* [cm^−1^]	*ω_LO_* [cm^−1^]	RB Width [cm^−1^]
AlN	671.34	910.50	239.16	615.00	879.40	264.40
Sc_0.04_Al_0.96_N	660.85	892.40	231.55	615.00	868.70	253.70
Sc_0.07_Al_0.93_N	660.52	885.60	225.08	615.00	862.00	247.00
Sc_0.10_Al_0.90_N	651.00	880.40	229.40	615.00	854.00	239.00
Sc_0.20_Al_0.80_N	636.80	862.70	225.90	635.00	770.00	235.00

## Data Availability

The original contributions presented in this study are included in the article. Further inquiries can be directed to the corresponding authors.
